# A population-based epidemiological study of anaphylaxis using national big data in Korea: trends in age-specific prevalence and epinephrine use in 2010–2014

**DOI:** 10.1186/s13223-018-0251-z

**Published:** 2018-07-02

**Authors:** Kyunguk Jeong, Jung-Dong Lee, Dae Ryong Kang, Sooyoung Lee

**Affiliations:** 1 Department of Pediatrics, Ajou University School of Medicine, Worldcup-ro 164, Yeongtong-gu, Suwon, 16499 Republic of Korea; 2 Office of Biostatistics, Ajou University School of Medicine, Worldcup-ro 164, Yeongtong-gu, Suwon, 16499 Republic of Korea

**Keywords:** Anaphylaxis, Epidemiology, Emergency, International classification of diseases

## Abstract

**Background:**

Previous reports on anaphylaxis in Asia are limited to relatively small-scale studies. We performed this study to identify the nationwide prevalence of anaphylaxis and epinephrine prescription rates by age groups.

**Methods:**

The total number of patients, yearly and overall prevalence, percentage of emergency department visits, and epinephrine prescription rates were calculated for patients diagnosed with anaphylaxis based on the Korean National Health Insurance database from 2010 to 2014.

**Results:**

The mean prevalence of anaphylaxis in Korea was 26.23 (95% confidence interval, CI 25.78–26.68) per 100,000 person-years during the 5 years. It increased from 20.55 (95% CI 20.15–20.10) in 2010 to 35.33 (95% CI 34.81–35.85) per 100,000 person-years in 2014. The average prevalence was > 35 per 100,000 person-years among 50–69 year-olds, and the mean crude prevalence in children was 22.3 (0–2 years), 17.3 (3–6 years), 12.1 (7–12 years), and 14.9 (13–17 years) per 100,000 person-years, respectively. The overall prevalence increased 1.7-fold, with the highest rate of increase in 0–2 years of age. The overall percentage of emergent anaphylaxis patients was 88.4%, and the prevalence of emergent anaphylaxis increased from 18.63 (95% CI 18.25–19.01) to 31.28 (95% CI 30.79–31.77) per 100,000 person-years. In-hospital epinephrine prescription rate increased from 31.5 to 39.7%.

**Conclusions:**

The mean prevalence of anaphylaxis in Korea was 26.2 per 100,000 person-years during the study period. The total number of anaphylaxis patients increased 1.7-fold from 2010 to 2014, with the most noticeable increment being in young children.

## Background

Anaphylaxis is an acute, potentially life-threatening hypersensitivity reaction [1]. According to a recent systematic review from Europe, the incidence rates for all-cause anaphylaxis ranged from 1.5 to 7.9 per 100,000 person-years, and approximately 1 in 300 of the European population experienced anaphylaxis at some point in their lives [2]. A significant increase in the occurrence of anaphylaxis has been reported in several parts of the world: a 615% increase in hospital admissions from all-cause anaphylaxis from 1992 to 2012 in the United Kingdom, a 1.5-fold increase in food-related anaphylaxis admission rates from 1998 to 2012 in Australia, and a 4.3% increase per year in the incidence rate of all-cause anaphylaxis from 2001 to 2010 in Olmsted County, Minnesota [3–5]. The rise in anaphylaxis incidence was more pronounced in children [3, 6–8]. Compared to copious reports on the epidemiology of anaphylaxis in Western countries, less is known about anaphylaxis in Asia, with most of the studies limited to retrospective case analyses [9–11]. Intramuscular epinephrine is the medication of choice for the first-aid treatment of anaphylaxis. Delayed administration of epinephrine is associated with poor outcomes including fatality; however, epinephrine is still under-prescribed [12–16]. A study from 15 university hospitals in Korea with adult anaphylaxis showed that only 30% received epinephrine [10].

The objective of our study was to evaluate the nationwide prevalence of anaphylaxis and epinephrine administration rates among anaphylaxis patients in Korea from 2010 to 2014 using a large nationwide administrative claims database, stratified by sex and various age groups, and location of presentation.

## Methods

### Case identification and validation of anaphylaxis

**Fig. 1:**
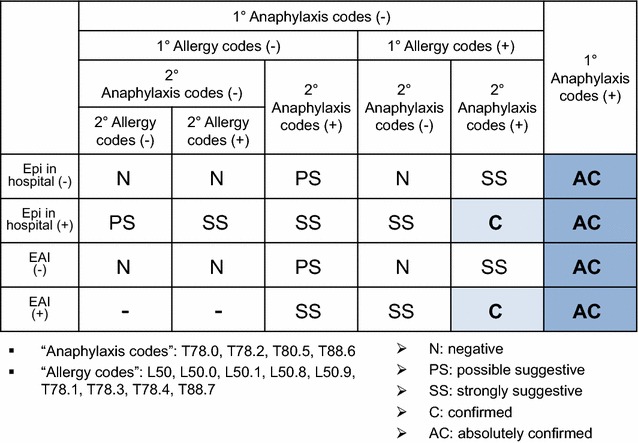
Operational definition of anaphylaxis

A population-based retrospective study was performed using the Korean National Health Insurance (NHI) database from the health insurance review and assessment service system. The NHI covers approximately 98% of the overall Korean population, and contains information including patient demographics, diagnostic codes, and prescriptions. Diagnostic codes are based on the Korean classification of diseases, 6th edition, which is equivalent to the 10th revision of the international classification of diseases (ICD-10). To minimize the possibility of under- or over-estimation of the study population, an “operational definition” using both diagnostic codes and prescription details was applied to assign the stepwise probability of anaphylaxis, from “absolutely confirmed” to “possibly suggestive” in decreasing order of possibility (Fig. 1).

This study included cases designated as “absolutely confirmed” and “confirmed”, excluding “strongly suggestive” and “possibly suggestive” to minimize over-estimation of anaphylaxis. From January 2010 to December 2014, cases with anaphylaxis-associated codes (T78.0: anaphylactic reaction due to food, T78.2: anaphylactic shock, unspecified, T80.5: anaphylactic reaction due to serum, and T88.6: anaphylactic reaction due to adverse effect of correct drug or medicament properly administered) as the primary diagnoses were included regardless of epinephrine prescription. In addition, cases with allergy-related codes other than anaphylaxis (L50: urticaria, L50.0: allergic urticaria, L50.1: idiopathic urticaria, L50.8: other urticaria, L50.9: urticaria, unspecified, T78.1: other adverse food reactions, not elsewhere classified, T78.3: angioneurotic edema, T78.4: allergy, unspecified, T88.7: unspecified adverse effect of drug or medicament) as the primary diagnosis and anaphylaxis-associated codes as the secondary diagnosis were included only if epinephrine was administered in hospital or epinephrine auto-injector (EAI) was prescribed.

### Statistical analysis

All data were analyzed using SAS Enterprise Guide 7.1 (SAS Institute, Inc., Cary, NC, USA). Categorical variables are presented as numbers and percentages. Age-specific annual prevalence rates of anaphylaxis from 2010 to 2014 were calculated by dividing the number of anaphylaxis patients by the Korean population from beneficiaries of health insurance and medical aid in 2010–2014 NHI statistical yearbooks. [17] A Poisson distribution was assumed for calculating 95% confidence intervals (CIs) for rates of prevalence. [18] The prevalence was calculated per 100,000 person-years for each age group. Among children and adolescents, the age groups were categorized as 0–2 years (infants and toddlers), 3–6 years (preschool children), 7–12 years (schoolchildren), and 13–17 years (adolescents), rather than typical 5-year intervals, to illustrate the epidemiological pattern according to developmental stages.

## Results

The number of patients in each category according to the operational definition of anaphylaxis is presented in Table 1. The number of patients in all categories showed an increasing trend during the 5 years from 2010 to 2014.

**Table 1:** Total number of patients according to probability of anaphylaxis

Year	Absolutely confirmed (AC)	Confirmed (C)	Strongly suggestive (SS)	Possibly suggestive (PS)
2010	9680	10,049	23,019	25,972
2011	10,126	10,610	24,633	27,786
2012	12,307	12,913	29,275	33,228
2013	13,016	13,745	31,311	34,813
2014	16,818	17,778	38,138	42,679

The numbers in the previous categories are included in the subsequent category (AC ⊂ C ⊂ SS ⊂ PS)

### Overall and age-specific prevalence of anaphylaxis

**Fig. 2:**
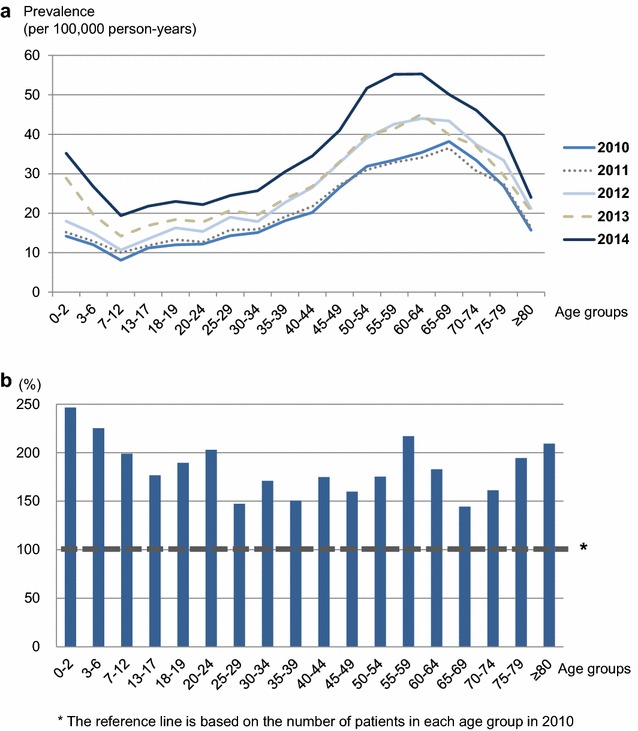
**a** Yearly prevalence of anaphylaxis by age group in 2010–2014. **b** Percentage increase of anaphylaxis patients by age groups in 2014 compared to 2010

The average overall prevalence of anaphylaxis during the study period was 26.23 (95% CI 25.78–26.68) per 100,000 person-years. The crude prevalence of anaphylaxis increased markedly from 20.55 (95% CI 20.15–20.95) per 100,000 person-years in 2010 to 35.33 (95% CI 34.81–35.85) per 100,000 person-years in 2014. As represented in Fig. 2a, the prevalence of anaphylaxis increased year by year from 2010 to 2014 in almost all age groups. The age-specific trend showed a decreasing trend from infants to schoolchildren, a gradually increasing trend from adolescents to middle ages, and finally a declining trend towards the older ages. The prevalence of anaphylaxis was considerably higher in the middle-aged groups from 45 to 65 years in each year of the study period. Among children and adolescents up to 18 years, the prevalence of anaphylaxis was markedly higher in the youngest age group (0–2 years) in each year of the study period. The overall prevalence of anaphylaxis increased by 1.7-fold during the 5 years, with the most noticeable increment being in young children (Fig. 2b).

### The number of anaphylaxis patients by sex

**Fig. 3:**
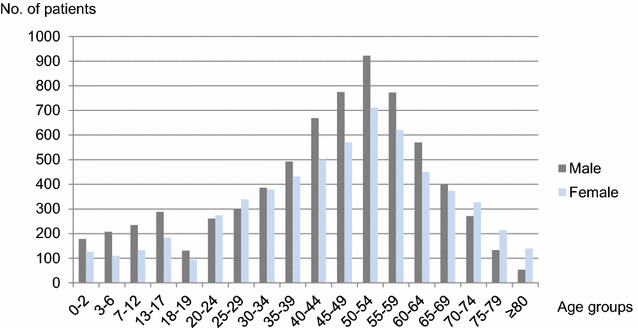
Average number of anaphylaxis patients by sex in 2010–2014

The average yearly numbers of anaphylaxis patients by sex during the study period are shown in Fig. 3. The number of male patients was higher in children and adolescents (< 20 years), partly in young adults and in middle-aged groups (30–69 years), whereas the number of female patients was higher in young adults in their twenties and old-age groups of 70 or older.

### “Emergent” anaphylaxis

**Fig. 4:**
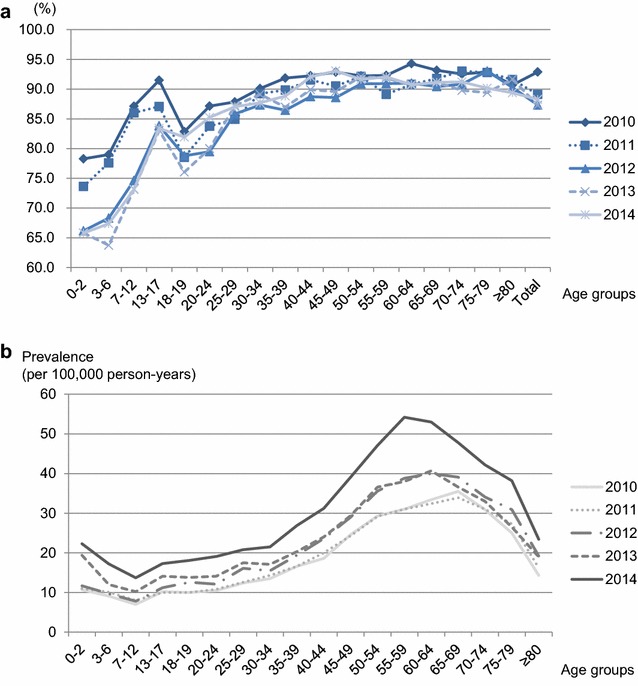
**a** Percentage of emergent anaphylaxis patients by age groups. **b** Yearly prevalence of emergent anaphylaxis patients by age group in 2010–2014

Among the total cases of anaphylaxis, “emergent” anaphylaxis patients were distinguished by identification of the route of hospital visit as emergency departments (EDs). The average percentages of emergent anaphylaxis patients in each year from 2010 to 2014 were 92.9, 89.1, 87.3, 87.3, and 88.3%, respectively. As shown in Fig. 4a, the proportion of emergent anaphylaxis patients was generally lower in younger age groups except adolescents. The proportion of emergent anaphylaxis showed a slightly decreasing trend during the 5 years, and the percentage declined markedly from 78.3 to 65.8% in the youngest age group of 0–2 years. Although the overall percentage of emergent anaphylaxis decreased slightly, the crude prevalence of emergent anaphylaxis increased, with a similar age-specific pattern to that of total anaphylaxis cases (Fig. 4b). The prevalence of emergent anaphylaxis increased year by year from 18.63 (95% CI 18.25–19.01) per 100,000 person-years in 2010 to 31.28 (95% CI 30.79–31.77) per 100,000 person-years in 2014. The prevalence of emergent anaphylaxis was generally higher in patients in their fifties and sixties and lower in children and adolescents. However, the increase in the prevalence of emergent anaphylaxis was greater in young children, specifically those in the 0–2 year and 7–12 year age groups.

### Epinephrine administration in hospital among anaphylaxis patients

**Fig. 5:**
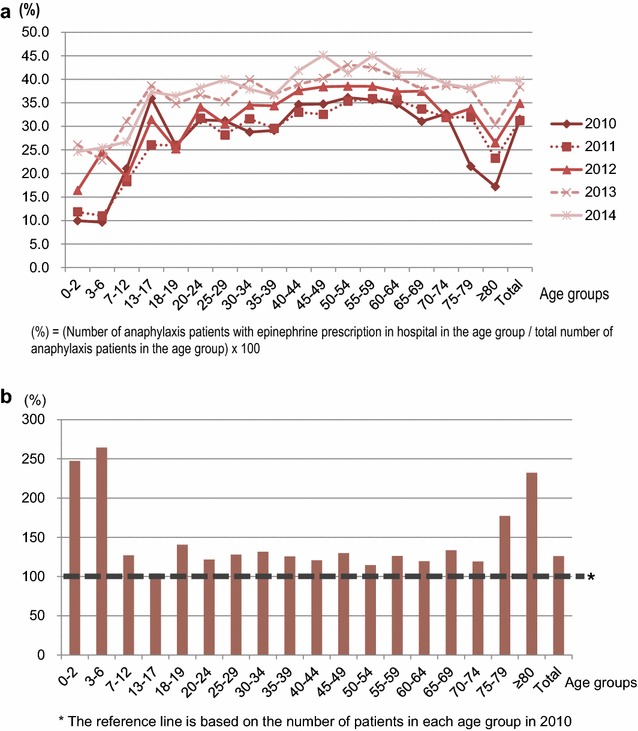
**a** In-hospital epinephrine administration among anaphylaxis patients. The percentages are calculated by dividing the number of anaphylaxis patients with epinephrine prescription in hospital in the age group by the total number of anaphylaxis patients in the age group, and then multiplying by 100. **b** Percentage increase of in-hospital epinephrine administration among anaphylaxis patients in 2014 compared to 2010

The average percentage of anaphylaxis patients administered epinephrine in hospital during the 5 years was 35.8%, and it increased from 31.5% in 2010 to 39.7% in 2014. As represented in Fig. 5a, the percentage of in-hospital epinephrine administration among anaphylaxis patients was approximately 10% in 0–6-year-olds, between 25 and 35% in young and middle-aged adults, and decreased to 17% in those aged 80 years and older. The age group with the highest average percentage of in-hospital epinephrine administration was 50–59 years. Compared to 2010, the percentage of in-hospital epinephrine administration in 2014 increased significantly, particularly in the younger age groups, by 2.5-fold in the 0–2 year and by 2.6-fold in the 3–6-year age groups (Fig. 5b). The percentage increase was also high in those aged 75 years and older.

### Epinephrine auto-injector prescription among anaphylaxis patients

Table 2 shows the number of EAIs among anaphylaxis patients in each year. All forms of the commercially available EAIs in Korea were included in this analysis (EpiPen^®^ 0.3 mg and EpiPen^®^ 0.15 mg from 2010–2014, Jext^®^ 0.3 mg and Jext^®^ 0.15 mg in 2014). The percentage of EAI prescriptions was as low as 2.4% in 2010, and subsequently showed an increasing pattern during the 5 years, reaching 6.9% in 2014. The percentage of EAI prescription was calculated by dividing the number of EAI prescriptions by the total number of anaphylaxis diagnoses and then multiplying by 100 for each year. For example, when the same patient visited hospitals more than twice due to anaphylaxis in the same year, it was separately counted and included in the denominator.

**Table 2:** Epinephrine auto-injector prescription in anaphylaxis patients

Year	EpiPen 0.3 mg/Jext 0.3 mg	EpiPen 0.15 mg/Jext 0.15 mg	Total
	N	N	N (%)
2010	242	54	296 (2.4)
2011	465	76	541 (3.8)
2012	631	180	811 (4.2)
2013	901	320	1221 (5.7)
2014	1378	523	1901 (6.9)

## Discussion

To our knowledge, this is the first population-based study to investigate the age-specific epidemiology of anaphylaxis in Korea, with tight intervals of age classification especially in children and adolescents. Furthermore, in-hospital epinephrine administration rates and EAI prescription rates were examined in this study.

In this study, the average prevalence of anaphylaxis peaked in the 60–64 year age group, whereas previously reported population-based studies identified various age groups as peak groups: the 30- to 39-year-old group in a 10-year Olmsted County study, and the 50- to 59-year-old group in a 7-year Korean study [5, 19]. As in many previous studies [4, 6, 8, 20], a remarkable increase in the overall prevalence of anaphylaxis was observed in this study, and the rather exceptional result in this study was that the increase was noticeably more pronounced in young children. The notable point of this study is that the age groups were categorized in tight intervals of 5 years in adults, and even narrower intervals in children and adolescents based on developmental stages. This specific categorization is meaningful because the prevalence of anaphylaxis showed a distinctive pattern between sub-groups among children and adolescents in this study. Moreover, the rate of increase in the prevalence of anaphylaxis was noticeable between the sub-groups during the 5-year study period. In 2010, the prevalence of anaphylaxis in the 0–2-year age group was 14.2 per 100,000 person-years, which was only slightly higher than that in the 3–6-year age group (12.0 per 100,000 person-years); however, the gap between the two groups widened significantly in 2014 (35.2 per 100,000 person-years in the 0–2-year age group versus 26.7 per 100,000 person-years in the 3–6-year age group). A previous study from the United States reported that the increase in anaphylaxis-related ED visits was greatest among children aged 5–17 years [21].

An analysis of anaphylaxis triggers was not included in this study because the diagnostic codes based on the healthcare claims are less likely to reflect the actual causes of anaphylaxis in clinical settings. While a previous Korean NHI data analysis reported that the subtypes of anaphylaxis were in order of unspecified (83%), food (10%), drug (6.6%), and serum (0.4%) based on ICD codes [19], hospital-based case studies revealed that drugs and foods were the most common triggers of anaphylaxis in Korean adults and children, respectively [9, 10]. Similarly, the disparity between a national data analysis and hospital-based case studies was also observed in other regions such as Europe [22, 23]. The population-based national data analysis has the advantage of being able to analyze parameters such as prevalence; however, it has a disadvantage in that reliable analysis of the detailed factors of anaphylaxis, such as triggers and symptoms, is rather difficult. Therefore, this study focused more on the epidemiological features and time trends of the disease rather than the detailed features of anaphylaxis. Further population-based study is needed for validation of nationwide anaphylaxis triggers based on an improved diagnostic code system in the future.

This study showed that there was a larger number of female than male anaphylaxis patients in the 20–29 year age group and in those aged 70 years or older. The prevalence of anaphylaxis was slightly higher in middle-aged females in several studies; however, the sex-related differences in anaphylaxis do not show a uniform pattern in previous studies except a male predominance in children and adolescents [5, 24]. A Spanish study reported higher anaphylaxis-related admission rates for males in general [8].

Among all anaphylaxis patients, the average percentage identified through ED visits from 2010 to 2014 was 88.4%, which was a comparable result to a previous report on food-induced anaphylaxis from the United States [25]. The percentage of emergent anaphylaxis patients decreased slightly from 92.9% in 2010 to 88.3% in 2014, and the decline was more pronounced in younger children, possibly due to the increased visits to outpatient allergy clinics for further investigations arising from increased awareness of anaphylaxis in the general population. Although the proportion of emergent anaphylaxis showed a slightly decreasing trend during our 5-year study period, the crude prevalence of anaphylaxis-related ED visits increased by 1.7-fold, as in several previous studies [21, 24]. This increment in the prevalence of anaphylaxis-related ED visits demonstrates a true rise of anaphylaxis in our region, because the patients presenting to the ED who met inclusion criteria for this study were more likely to have had anaphylaxis on the day of their visit (in comparison to those who presented in outpatient settings). Therefore, the increase in emergent visits is more likely reflective of an overall increase in anaphylaxis in Korea, which is attributed to both the increase in the disease itself and the increased awareness of the public and the medical staff. Especially, the greater increment in the emergent anaphylaxis in children is this study is likely to be associated with an increasing food allergy itself, as food is the most common cause of anaphylaxis in pediatric age. A recent Canadian study of anaphylaxis in pediatric EDs also reported that food was mostly responsible [26]. This significant increase in emergent anaphylaxis emphasizes the importance of the ED as the front line for the diagnosis and treatment of anaphylaxis, and therefore its significance as a future research setting in the study of anaphylaxis.

The average percentage of in-hospital epinephrine administration among total anaphylaxis cases in our study was 35.8%, which was relatively low but comparable to previous studies. A previous study of infants from 3 to 15 months also reported a low epinephrine prescription rate of 29.9%, and a Taiwanese study revealed that only 2.2% of anaphylaxis patients received epinephrine [11, 25]. Despite the universal recommendation of prompt epinephrine use in anaphylaxis, possible reasons for not administering epinephrine include lack of recognition of anaphylaxis symptoms, epinephrine being unavailable, and concerns related to adverse effects of epinephrine. The prescription rate of EAIs among anaphylaxis patients was relatively low in our study, though there was a 2.8-fold increase from 2.4 to 6.9% in the EAI prescription rate during our 5-year study period. Comparison of the proportion and trend of EAI prescriptions with previous studies is rather difficult because population-based analysis of EAI prescription rates among anaphylaxis patients remains rare. The Olmsted study reported that the prescription rates of EAIs per patient per year increased from 2004 to 2010, with an annual increase rate of 8%, and the rate of EAI prescriptions was 7.4 and 7.7% in a multicenter retrospective case study of anaphylaxis in Korean adults and a questionnaire-based study among Mexican schoolchildren with food-induced anaphylaxis, respectively [10, 27, 28]. An age-specific analysis of EAI prescriptions was not performed in this study because the yearly numbers of EAI prescriptions were not sufficient for statistical analysis by age groups.

Although the present study was comprehensive, it is not without limitations. First, the possibility of inaccurate coding or misclassification exists because studies based on administrative data rely heavily on the system of diagnostic codes as well as physicians’ diagnostic code input behavior. To minimize over- or under-detection of “true anaphylaxis” and maximize the accuracy of our analysis, we used the operational definition using both the order of diagnostic code and prescription of in-hospital and/or EAI. The second limitation is that analysis of the details of anaphylaxis cases such as triggers, symptom profiles, or severity was not allowed due to the nature of national data. To improve accuracy in the future, the disease classification system needs to be further refined to account for the triggers and severity of anaphylaxis.

However, the strength of the present study is that this is the first population-based study of anaphylaxis to focus on age-specific prevalence including anaphylaxis-related ED visits, as well as epinephrine prescriptions, including EAIs. The remarkably high increase of the anaphylaxis prevalence in young children < 2 years, and the nationwide age-specific epinephrine prescription rates were novel findings of our study. In future, more detailed big data analysis of anaphylaxis such as main triggers of anaphylaxis would be possible if the diagnosis code related to anaphylaxis is more systemized. We also expect more comprehensive analysis by linking national big data and hospital-based data.

## Abbreviations

CI: confidence interval
EAI: epinephrine auto-injector
ED: emergency department
ICD: international classification of diseases
